# Non-invasive neuromodulation effects on painful diabetic peripheral neuropathy: a systematic review and meta-analysis

**DOI:** 10.1038/s41598-020-75922-9

**Published:** 2020-11-05

**Authors:** Huiyan Zeng, Kevin Pacheco-Barrios, Ying Cao, Ying Li, Jinming Zhang, Caifeng Yang, Felipe Fregni

**Affiliations:** 1 Department of Endocrinology, The Second Affiliated Hospital of Guangzhou University of Chinese Medicine, 261 Datong Road, Er Sha Island, Guangzhou, 510105 China; 2 Neuromodulation Center and Center for Clinical Research Learning, Spaulding Rehabilitation Hospital and Massachusetts General Hospital, Harvard Medical School, 96 13th Street, Charlestown, Boston, MA USA; 3 Unidad de Investigación Para La Generación Y Síntesis de Evidencias en Salud, Vicerrectorado de Investigación, Universidad San Ignacio de Loyola, Lima, Peru; 4 Department of Endocrinology, Nanfang Hospital, Southern Medical University, Guangzhou, Guangdong China

**Keywords:** Peripheral neuropathies, Diabetes complications

## Abstract

Diabetic Peripheral Neuropathy (DPN) typically is accompanied by painful symptoms. Several therapeutic agents have been tried for symptomatic relief, but with varying results. The use of non-invasive neuromodulation (NINM) is a potential treatment option for DPN. The objective of our study is to evaluate NINM effects on pain rating and nerve conduction velocity in DPN patients. The search was carried out in seven databases until Aug 30th, 2019. Finally, twenty studies met the inclusion criteria. We found a significant reduction of pain scores by central NINMs (effect size [ES] =  − 0.75, 95% CI =  − 1.35 to − 0.14), but not by the overall peripheral techniques (electrical and electromagnetic) (ES =  − 0.58, 95% CI =  − 1.23 to 0.07). However, the subgroup of peripheral electrical NINMs reported a significant higher effect (ES =  − 0.84, 95% CI =  − 1.57 to − 0.11) compared to electromagnetic techniques (ES = 0.21; 95% CI =  − 1.00 to 1.42, I^2^ = 95.3%) . Other subgroup analysis results show that NINMs effects are higher with intensive protocols and in populations with resistant symptoms or intolerance to analgesic medications. Besides, NINMs can increase motor nerves velocity (ES = 1.82; 95% CI = 1.47 to 2.17), and there were no effects on sensory nerves velocity (ES = 0.01, 95% CI =  − 0.79 to 0.80). The results suggest that central and peripheral electrical NINMs could reduce neuropathic pain among DPN patients, without reported adverse events. Well-powered studies are needed to confirm that NINM techniques as an alternative effective and safe treatment option.

## Introduction

The prevalence of diabetes mellitus continues to increase alarmingly, and it is estimated that nearly half a billion people are living with diabetes worldwide in 2019^1^. Approximately 50% of patients develop peripheral neuropathy due to diabetes^2^. It is one of the most common complications of diabetes mellitus. Diabetic peripheral neuropathy (DPN) usually affects the sensory, motor, and autonomic nervous systems^3^. Also, it is typically accompanied by extremely painful symptoms, including tingling, burning, sharp, shooting, or an electric shock sensation, which often presents nocturnal exacerbation. Such pain has a considerable impact on the patient's quality of life by leading to sleep disturbances, anxiety, depression, loss of mobility, and independence^4^. Besides, advanced neuropathic deficits underlie most foot ulceration and amputation, gait disturbance, and fall-related injury in diabetic patients^5^.

Painful DPN remains a major therapeutic challenge. There are several treatment options as analgesics, antiepileptic drugs, antidepressants, and antioxidants, but effects vary^6^. In addition to the efficacy variability, some are related to adverse events as toxicity, drug abuse, or gastrointestinal events^6^. This situation has created interest in alternative safe and effective non-pharmacological therapeutic strategies for painful DPN, and many neuromodulation approaches have been proposed^7^.

In the past years, non-invasive neuromodulation (NINM) techniques have been increasingly used in the treatment of pain^8,9^. It has been showed that this neurotechnology is a feasible and safe treatment for chronic neuropathic pain^10,11^. NINM interventions can be divided into central and peripheral neurostimulation. The most studied central NINM techniques include repetitive transcranial magnetic stimulation (rTMS) and transcranial direct current stimulation (tDCS)^12^, and the transcutaneous electrical neural stimulation (TENS) is the most common peripheral NINM technique^13,14^. tDCS delivers a subthreshold current from anode to cathode by two electrodes over the scalp, whereas rTMS uses magnetic fields in order to induce electrical changes in the brain activity^15^. Similar principle is applied by TENS, it uses an electrical current to modify the membrane polarity of peripheral nerves^16^. All of these techniques can modulate brain function through inducing changes in polarity of the neuronal membrane^12,17^ and based on the Melzack and Wall’s neuromatrix theory of pain^18^, they could modulate pain perception restoring the balance in the inhibitory endogenous pain pathways regulation while preventing or reversing maladaptive plasticity leading to a decrease of pain^15^.

Recently, publications on NINM techniques as a treatment for painful DPN grew. Previous narrative^19,20^ and systematic reviews^21–23^ have addressed the use of NINM techniques as therapeutic option for chronic pain syndromes reporting small to moderate positive effect sizes, however, those studies included some patients with DPN, but failed to study specifically the DPN population; furthermore, they did not compare central versus peripheral techniques or include changes on neurophysiological biomarkers such as nerve conduction studies, which represents axonal and myelin damage and could be use as biomarkers of nerve damage recovery in DPN patients^24^. No systematic reviews summarizing the use of NINM techniques in this condition are reported; hence, the efficacy and safety of these techniques on patients with painful DPN and their effects on neurophysiological biomarkers remain unclear. Our systematic review aims to evaluate the NINM effects on pain intensity reduction and nerve conduction velocity (NCV) improvement in adult individuals with painful DPN.

## Results

### Overview

**Figure 1:**
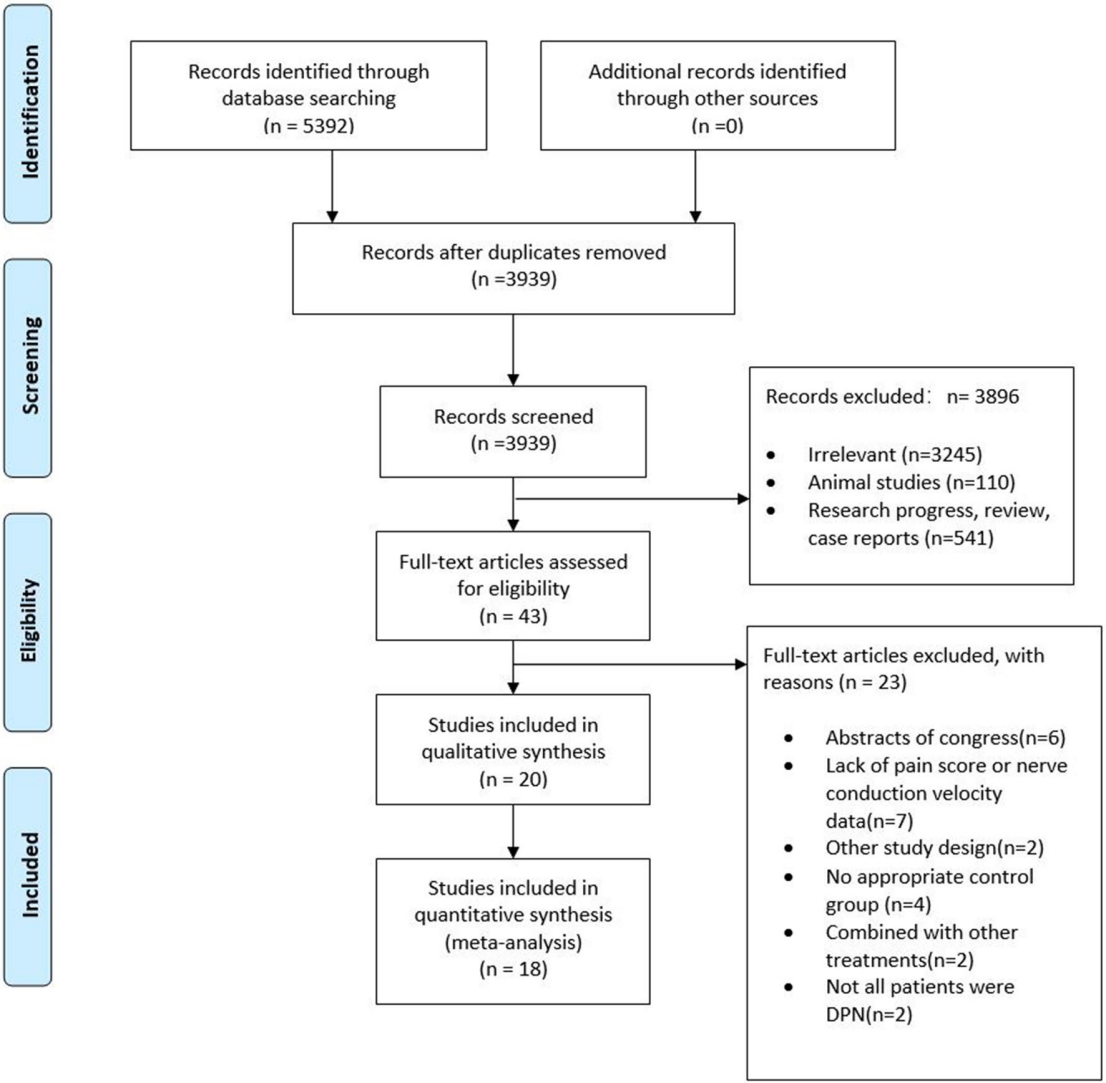
PRISMA flow diagram (study selection).

A total of 5392 articles were retrieved through the search strategy, and 3939 remained after removing duplicates, of which 3896 were excluded based on the title and abstract. Twenty trials (18 randomized-controlled trials [RCTs] and 2 Quasi-experiment [QE]) with 1167 patients were included from 43 potentially relevant publications after evaluating the full-text. A flow diagram of the searched and evaluated literature is presented in Fig. 1. We included studies on seven NINMs techniques: rTMS, tDCS, “mesodiencephalic” modulation (MDM), TENS, percutaneous electrical nerve stimulation (PENS), pulsed electromagnetic field therapy (PEMF), and frequency-modulated electromagnetic neural stimulation (FREMS). We classified those interventions in (1) central (3 studies: rTMS, tDCS, and MDM) and (2) peripheral techniques (17 studies: also subdivided in electrical [TENS and PENS] and electromagnetic [PEMF and FREMS] peripheral techniques). Descriptive information of the studies' characteristics of each article included is provided in Table 1 and Supplementary Material 4.

**Table 1:** Summary of included studies characteristics.

Study	Study Design	Intervention	N (total)	Use of analgesics	Outcomes
Onesti et al.^36^	Double Crossover	Active rTMS versus sham	23	YES	VAS(0–100); RIII reflex
Weintraub et al.^38^	RCT	PEMF versus sham	194	YES	VAS (0–10); NPS(0–100); PGIC
Zahra and Serry^42^	RCT	TENS versus exercise versus pharmacological group	60	YES	VAS; sensory NCS
Oyibo et al.^37^	Crossover	PENS versus sham	14	NO	VAS
Naderi Nabi et al.^35^	RCT	PRF versus TENS	60	YES	NRS
Kumar et al.^32^	RCT	PENS vs sham group	31	NO	Pain grade
Kim et al.^30^	RCT	M1 tDCS versus DLPFC tDCS vs sham	60	YES	VAS; CGI score; anxiety score; sleep quality; BDI; PPT
Hamza et al.^29^	Crossover study	PENS versus sham	50	YES	VAS; SF-36; BDI; POMS
Gossrau et al.^28^	RCT	TENS versus sham	40	NO	PDI; NPS; CES-D
Lacigová et al.^33^	cross-over study	MDM versus sham	60	YES	VAS; TSS; BDI; SF-36
Kumar et al.^31^	RCT	PENS versus sham	23	YES	Pain grade
Abdelkader et al.^43^	Quasi-experiment	rTMS in both groups: insulin dependent versus non-insulin dependent group	20	NO	VAS
Moharič et al.^34^	RCT	TENS versus pregabalin versus combined group	65	YES	VAS; SF-36
Bulut et al.^26^	RCT	TENS versus sham	40	NO	VAS; Pain grade
Yuanhong Ding et al.^41^	RCT	TENS versus pharmacological group	60	YES	MCV; SCV; Pain grade
Yonghong Guo et al.^40^	RCT	PENS versus pharmacological group	68	YES	MCV; SCV; hemorheology
Wróbel et al.^39^	RCT	PEMF versus sham	61	YES	VAS; EuroQol EQ-5D; MOS; Sleep Scale
Armstrong et al.^44^	Quasi-experiment	PENS	10	NO	VAS
Bosi et al.^25^	Crossover	FREMS versus sham	31	NO	VAS; MCV; SCV; SF36; VPT
Forst et al.^27^	RCT	TENS versus sham	19	NO	NTSS-6; VAS

DN4: Douleur Neuropathique 4 Questions; rTMS: repetitive transcranial magnetic stimulation; VAS: Visual analog scale; NPS: Neuropathy Pain Scale; PGIC: Patient’s Global Impression of Change; PI-NRS: Pain Intensity Numerical Rating Scale; PEMF: pulsed electromagnetic field therapy; TENS: transcutaneous electrical nerve Stimulation; NDS: neuropathy disability score; PENS: percutaneous electrical nerve stimulation, PRF: Pulsed Radiofrequency Sympathectomy; FREMS: frequency-modulated electromagnetic neural stimulation tDCS: transcranial direct current stimulation; M1: primary motor cortex; DLPFC: dorsolateral prefrontal cortex; CGI: Clinical Global Impression; BDI: Beck Depression Inventory; PPT: pressure pain threshold; NTSS: neuropathy total symptom score; POMS: the Profile of Mood Status; SF-36: the MOS 36-Item Short-Form Health Survey; CES-D: Center for Epidemiologic Studies Depression Scale; IFT: Interferential therapy; MDM: “mesodiencephalic” modulation; MCV: motor conduction velocity; SCV: sensory conduction velocity; SEP: Somatosensory-evoked potential; PSP: Acupoint skin pain threshold; NIS LL: Neuropathy Impairment Score Low Limbs scale.

### Risk of bias assessment

**Figure 2:**
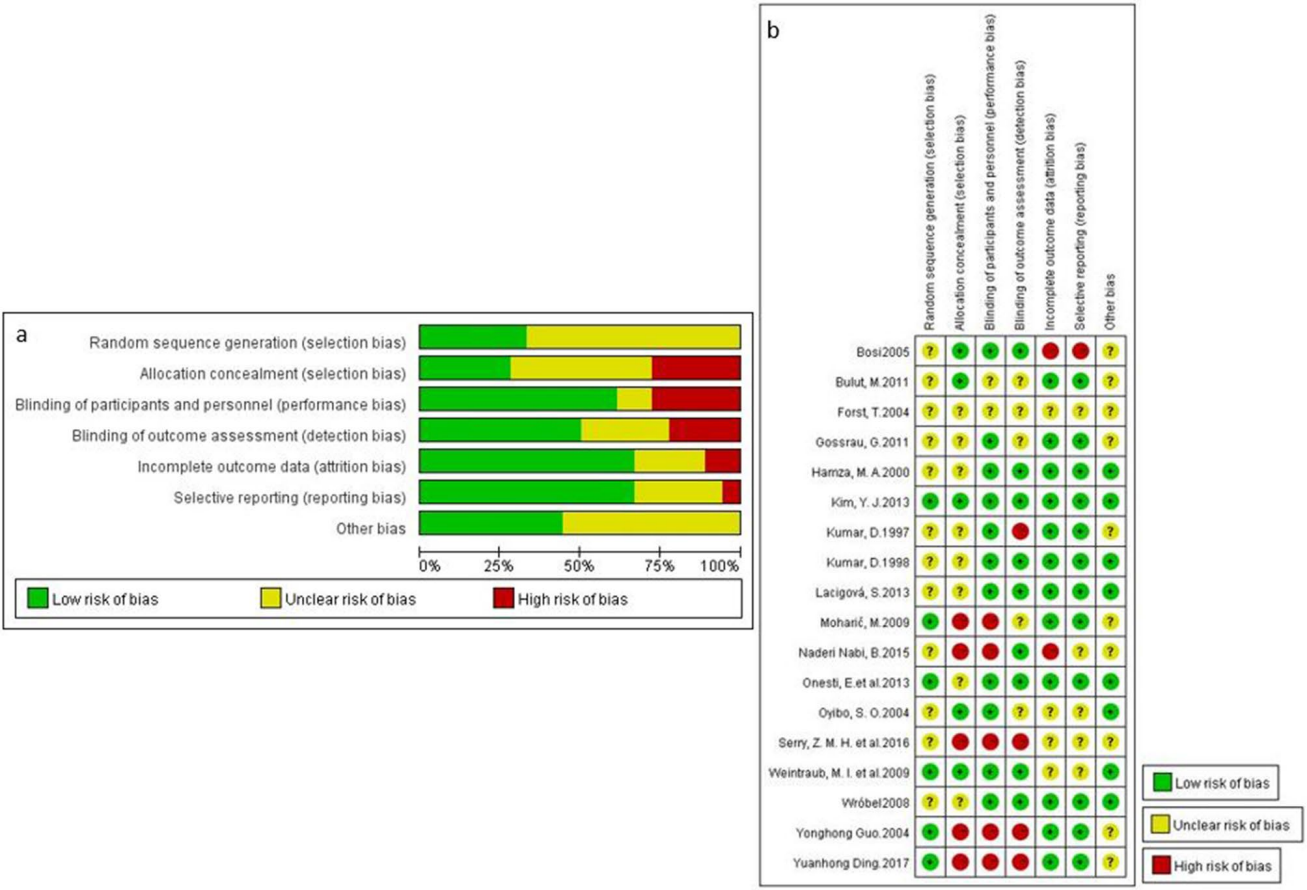
(**a**) Risk summary of bias of RCTs. (**b**) Risk details of bias of RCTs.

The risk of bias for individual 18 RCTs^25–42^ was assessed using the Cochrane tool. All the RCTs included mentioned that they used a randomization technique, but 12 (67%) of them lack information on how the sequence was generated. Most of the RCTs did not specify the allocation concealment (72%), blinding outcome assessment (50%), and personnel (39%) details, even though they described their design as blinded (including double-blinded and single-blinded design) (Fig. 2a,b.).

The risk of bias for individual two QE study^43,44^ was assessed using the Methodological index for non-randomized studies (MINORS) tool. The overall MINORS score of these two studies was 19 for Abdelkader 2019^43^ (low-risk) and 12 for Armstrong 1997^44^ (high-risk). In Abdelkader et al. study, the baseline characteristics between groups were not balanced. Besides, both studies did not report information about endpoint and unbiased assessment.

We did not find publication bias across the RCTs included—symmetrical funnel plot and non-significant Egger’s test (*p* = 0.417) (online supplementary material 6).

### Evidence from randomized-controlled trials

#### Effects on pain outcomes

**Figure 3:**
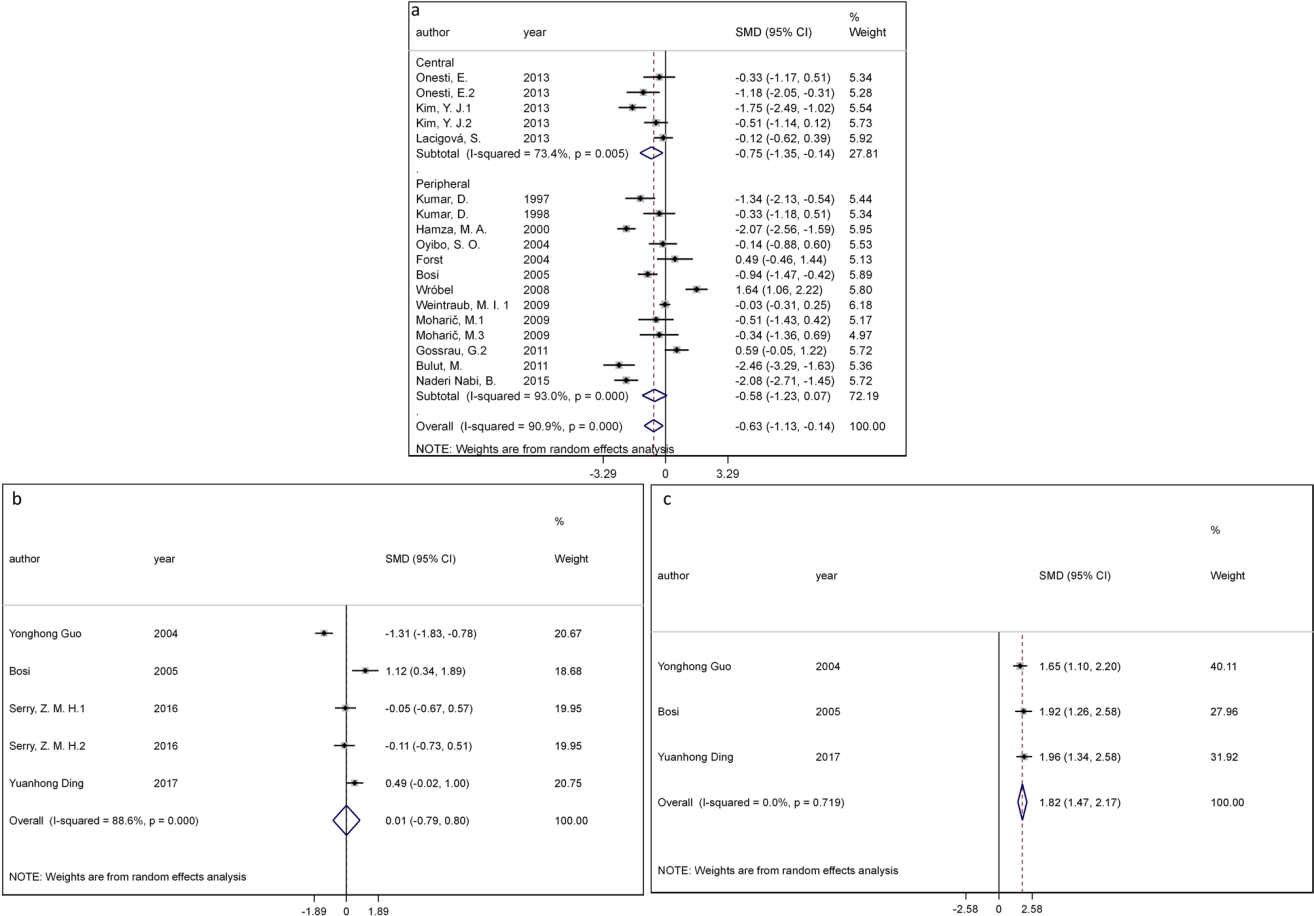
Meta-analysis results for (**a**) pain changes—overall techniques; (**b**) SCV changes—peripheral techniques; (**c**) MCV changes—peripheral techniques. All outcomes are shown compared with sham NINM or not NINM group with 95% confidence intervals.

The included studies evaluated the effect of NINMs in painful DPN patients by reporting pain reduction outcomes (14 studies^26–39^), NCV outcome (three studies^40–42^), and both of them (one study^25^). Pain score data were extracted from 15 RCTs (three studies testing central techniques and 12 testing peripheral interventions), which involved 909 patients in total, including 350 patients who were resistant or intolerant to analgesics (baseline pain score: 5.66 ± 1.50), 296 patients who were responders to analgesics (baseline pain score: 5.46 ± 1.72) and 263 patients who had no analgesics related information (baseline pain score: 5.76 ± 1.11). The baseline pain scores were not significantly different across these 3 subpopulations (ANOVA test, *p* = 0.54). From a random-effects model meta-analysis, we found a significant higher reduction of pain score by central NINM interventions (effect size [ES] =  − 0.75, 95% CI =  − 1.35 to − 0.14, I-squared statistic [I^2^] = 73.4%), but not by the overall peripheral techniques (including electrical and electromagnetic interventions) (ES =  − 0.58, 95% CI =  − 1.23 to 0.07, I^2^ = 93.0%) (Fig. 3a). However, the electrical peripheral techniques showed a higher effect size (ES =  − 0.84; 95% CI =  − 1.57 to − 0.11, I^2^ = 89.6%) (*p* = 0.001) compared to the electromagnetic (ES = 0.21; 95% CI =  − 1.00 to 1.42, I^2^ = 95.3%) (online supplementary material 5). The electrical peripheral techniques subgroup included studies with TENS (seven out of 12), predominantly.

Subgroup results showed that effects are higher (*p* = 0.002) and more precise in the studies with more intensive NINMs protocols (≥ 3 sessions per week) (ES =  − 0.61; 95% CI =  − 1.10 to − 0.12) compared with less intensive protocols (ES =  − 0.75; 95% CI =  − 3.36 to 1.86), although the less intensive protocols had a large point estimate, the confidence interval included the null value invaliding its interpretation. Besides, the NINM effects were significantly higher (*p* = 0.000) in studies with resistant or intolerant population (ES =  − 1.08, 95% CI =  − 1.60 to − 0.56), compared to analgesic responder population (ES =  − 0.20, 95% CI =  − 1.85 to 1.44). We did not find a difference with studies with sham-intervention versus other types of control groups. Only four RCTs (2 used central techniques and the rest used peripheral) have reported the more than 1-month follow-up effects of NINM showing still lasting analgesic effect (ES =  − 1.38, 95% CI =  − 2.22 to − 0.53) (online supplementary material 5). No adverse events were reported in the included studies.

#### Effects on NCV outcomes

The NCV data were available in only studies testing peripheral stimulation techniques. The peripheral NINMs interventions showed a significant and homogenous positive effect on motor conduction velocity (MCV) improvement (ES = 1.82; 95% CI = 1.47 to 2.17, I^2^ = 0.0%) but the effects on sensory conduction velocity (SCV) changes were not significant (ES = 0.01, 95% CI =  − 0.79 to 0.80, I^2^ = 88.6%) (Fig. 3b,c). Due to insufficient SCV and MCV data in the included studies, we only did subgroup analysis for pain rating outcomes.

#### Sensitivity analysis and meta-regression

In the sensitivity analysis, we evaluated the pooled effect changes by removing one study at the time for the central and peripheral analgesic effects, and for the NCV outcomes. We did not find large effect changes, demonstrating a consistency of our pooled effects estimates. Additionally, we did not find a difference in the effects considering the risk of bias category of the included studies.

Meta-regression was conducted on overall analgesic efficacy in DPN patients, demonstrating no significant influence of a number of sessions on the pooled effect estimated (*p* = 0.2). Also, we evaluated other sources of heterogeneity on the analgesic efficacy of peripheral electrical stimulation; we did not find a significant influence of stimulation frequency (*p* = 0.6) and treatment duration in days (*p* = 0.26).

### Evidence from quasi-experimental studies

We included two QE studies, we decided to keep separate from the RCT analyses to reduce the bias of our pooled estimates. These two studies^43,44^ reported non-combinable data, thus we could not perform a meta-analysis. One study (n = 20) used a central technique (high-frequency rTMS over motor cortex)^43^, the authors reported a pain reduction after intervention of 4.0 to 5.10 VAS points and a significant increase on MCV. The second study (n = 10) used an electrical peripheral intervention (PENS)^44^ and reported a 5.5 VAS points reduction after intervention, no data on NCV was available.

## Discussion

### Summary of results

We included 20 studies that have evaluated the effects of NINMs on pain score or/and nerve conduction velocity in painful diabetic peripheral neuropathy adults. These studies were heterogeneous with small sample sizes, but publication bias was not founded. We found a consistent medium to large effect size on pain reduction by central techniques, but no significant effects for the overall peripheral techniques, although we found a large effect for one of its subgroups (electrical peripheral techniques, predominantly TENS). Moreover, we found a big effect size of peripheral techniques on MCV improvement. No adverse events were reported in the included studies. The intensive stimulation protocols (≥ 3 sessions per week) and the inclusion of a resistant or intolerant population are the main sources of heterogeneity.

### Comparison with other studies

Jin et al.^45^ conducted a systematic review trying to evaluate similar question to ours, however, they only focused on one type of peripheral NINM technique—compared with the seven techniques included in the present review—and included only three studies (n = 78). The study evaluates the effectiveness of transcutaneous electrical nerve stimulation (TENS)—a type of electrical peripheral NINMs—on DPN patients. They found that TENS therapy significantly improved generally neuropathic pain symptoms in the treatment of symptomatic DPN (ES =  − 1.01, 95% CI − 2.01 to − 0.01). The authors concluded that TENS therapy is effective for painful DPN. Yet their results are consistent with ours; we reported an updated evidence body with a significant higher number of included studies and participants (eight studies, n = 378). Furthermore, NINMs effects seem to have a sustained pain reduction effect even when the intervention session had been finished more than 1 month. However due to the small number of included studies (5 studies, n = 265) our estimates have to be interpreted with caution, similar to other studies in the neuromodulation field where the absence of long term follow up hinders the clinical interpretation of the results^46,47^.

No previous systematic review of central NINMs techniques was found. Thus, our study is the first to evaluate these interventions. Our results (significant reduction of pain) are consistent with previous literature on tDCS and rTMS effects on other chronic pain^48^ and neuropathic pain conditions^46^, also the parameters used in our included studies are similar to previous studies, using motor cortex as the main target region with excitatory stimulation polarity^46^. But our results show a moderate to big effect compared with the small effects reported in other chronic pain conditions^46^. However, a final positive statement for the effects of central NINMs on pain cannot be issued due to the limited number of studies and the absence of long-term follow-up.

### Sources of heterogeneity and potential explanations

According to our subgroup analysis, central NINM techniques can reduce pain effectively in DPN patients compared with overall peripheral techniques. We hypothesized that central NINMs are accurately targeting the mechanism of neuropathic pain generation in diabetes. More and more evidence indicated that DPN does not influence peripheral nerves only, but also central structures involved in pain generation and perpetuation. For instance, cortical reorganization, central hypersensitization, neurotransmitter imbalance, and abnormal functional connectivity^49,50^. Lower brain grey matter volume (localized in primary somatosensory cortex, supramarginal gyrus and cingulate cortex) and correlation with neuropathy severity in DPN subjects had been proved in previous studies^51^. A multimodal clinical neuroimaging study had found that the functional connectivity of periaqueductal gray subregions^52^, and neurotransmitter imbalance (higher glutamate levels and less gamma-aminobutyric acid (GABA))^53^ is altered in diabetic patients with neuropathic pain. In addition, central sensitization (indexed by greater blood-oxygen-level-dependent [BOLD] responses of the primary somatosensory cortex, lateral frontal, thalamus, and cerebellar regions) is present in DPN patients with pain compared with those without pain^54^. Regarding the analgesic mechanism of NINMs, it had been confirmed that the distant circuits modulated by this type of technique, such as the cingulate cortex and thalamus, are crucial for the processing of pain in DPN^12,55,56^. A study found that anodal M1-tDCS reduces central sensitization-induced hyperalgesia through the descending pain modulatory network in humans^57^. The effects of tDCS on neurotransmitters had been detected by magnetic resonance spectroscopy and showed that anodal tDCS reduces local concentrations of the inhibitory neurotransmitter GABA, whereas cathodal tDCS reduces excitatory glutamate levels^58,59^. Furthermore, tDCS has been suggested that it may interfere with functional connectivity, synchronization, and oscillatory activities in various cortical and subcortical pain networks in some studies^60–63^. Taken together, we hypothesize that central NINMs could help DPN subjects to relieve pain through a change in the cortical reorganization; that ultimately enhances the inhibitory drive and thus reduce pain-related excessive excitability^64–68^.

Regarding the NCV outcomes, the effects of NINMs on SCV and MCV were in a different direction. NINM techniques increased MCV significantly but have no impact on SCV outcome. The number of studies reporting MCV outcomes was three; only one of them^25^ reported both significant increases of MCV and reduction of pain. This findings are consistent with the hypothesis of increasing peripheral input (due to peripheral nerve stimulation or other behavioral intervention such as exercise or motor tasks) can activate the sensorimotor cortex region (indexed by increases in intracortical facilitation and motor conduction velocity) and modulate its connection with thalamus reducing the maladaptive pain perception (leading to pain ratings reduction) by activating the inhibitory endogenous pain system^57,69^.

Regarding the NINMs protocols characteristics, our findings suggest that the intensive protocols (≥ 3 sessions per week), but not the number of total sessions (evaluated by meta-regression analysis), influence our pooled estimate of pain reduction. It seems that repeated NINM sessions over a period of time is more effective than doing the same number of sessions but with more extended time intervals. However, the limited range of the number of sessions and the overall short-term follow-up, we cannot provide any conclusion about the dose–response of NINMs intervention in DPN patients. Further studies comparing different stimulation protocols (escalated doses/sessions and variable time intervals among sessions) will be needed to confirm these findings.

Even though half of the included studies did not clarify the status of patients’ analgesic response to medications, the neuromodulation effects seem to be higher on the intractable DPN population. It has been reported that patients with intractable pain disorders have less treatment expectancy and placebo effect^70^. Consequently, we can argue that our pooled estimated effect is less likely to be affected by the placebo effect of the sham intervention. For intractable DPN population, there are limited effective therapeutic options to relieve pain such as spinal cord stimulation^71–74^, although its promising results, the cost and potential risk of the surgery procedure reduce the availability for DPN patients worldwide, therefore, the most common clinical approach includes intensive control of blood glucose and lifestyle change^63^. Sometimes, the neuropathic symptoms remain even when good glycemic control and lifestyle modifications has been achieved. Our findings support the potential clinical use of non-invasive neuromodulation techniques on intractable DPN patient groups. Hence, we suggest better designed multicenter RCTs on this population using non-invasive neuromodulation techniques.

## Limitations and strengths

Due to the heterogeneous stimulation parameters in the studies that were analyzed, it can be argued that this meta-analysis is not comparing similar interventions. However, since summarized effect estimates are needed for decision-making—to guide future research and clinical applications—we found meta-analyses useful to give a better overview of the results. Besides, the present meta-analysis has recognized limitations in the primary studies, as insufficient detail about the outcome evaluation, stimulation parameters, and what did the control group receive.

However, this study has important strengths: it followed the PRISMA statement and the Cochrane manual. In addition, we performed a comprehensive search strategy across multiple databases without language restriction. These strengths allow us to report state of the art on the research in non-invasive neuromodulation interventions in DPN and an exploration of the efficacy and safety of these interventions.

## Conclusion and research recommendations

Meta-analytic results suggest a significant medium to a large effect on neuropathic pain reduction among DPN patients by central NINMs techniques, and the subgroup of peripheral electrical interventions, without any adverse events reported. For patients with resistant symptoms or intolerance to analgesic medications, NINMs techniques could be an alternative safe treatment option. Due to the limited sample size and lack of follow-up sessions, more evidence is required before treatment recommendations can be made.

## Methods

A systematic review of the literature and meta-analysis was conducted following the recommendation of the Cochrane handbook^75^, including the PRISMA guidelines (online supplementary material 1)^76^.

### Literature search and study selection

We performed a search in eight databases (Medline, Web of Science, Scopus, Cochrane Central, Lilacs, Embase, Pedro, and China National Knowledge Infrastructure database) until September 30th, 2019 using the following MeSH terms: "noninvasive neuromodulation stimulation" OR "transcranial magnetic stimulation" OR "transcranial direct current stimulation" OR "transcutaneous electrical nerve stimulation" AND "diabetes" OR "diabetic" AND "neuralgia" OR "Chronic pain” OR "Neuropathic pain". The complete search strategy is shown in the online supplementary material 2.

We eliminated duplicates before selection process. Then, two reviewers (YC and JZ) agreed on a standard approach. After this standardization process, the titles and abstracts were independently screened by two reviewers (YC and JZ). A third reviewer (YL) solved any discrepancies between reviewers. Afterwards, the two reviewers independently assessed full texts of selected studies to confirm the eligibility criteria, a third reviewer solved any discrepancies.

### Eligibility criteria

We searched for full-text articles without language restrictions (only articles in English and Chinese were found). Included articles had to: (a) enroll adult DPN subjects; (b) performed NINM including tDCS, rTMS and TENS; (c) pain rating, pain score, symptoms score or NCV as assessment tools; (d) randomized controlled trials (RCTs), included parallel-group and crossover designs and pilot studies; and quasi-experimental trials, included non-controlled, non-randomized and one arm studies. A detail description of eligibility criteria is shown in Table 2.

**Table 2:** Eligibility Criteria for Considering Articles for the Review.

	Inclusion	Exclusion
Participants	DPN patients, over 18 years old	Diabetes patient with neuropathy caused by other reasons
Intervention	Studies that applied NINM, including TENS, tDCS, rTMS or other, as an intervention method	Research that presented results of NINM associated with other interventions (such as analgesic medications, not counting the basic therapy that use in both intervention group and control group)
Comparison	Studies in which the control group received sham NINM stimulation or no NINM stimulation	Studies with no placebo or blank control group
Outcome	(i) Pain intensity that measured before and after intervention by VAS or other pain score questionnaire; (ii) Nerve conduction velocity that measured before and after intervention	
Trial Design	(i)Randomized controlled clinical trials or crossover	
Studies; (ii) Quasi-experiments	Other study design, such as retrospective study and case–control study	
Type of Publication	Published in a peer-reviewed journal; regardless of the year of publication; regardless of language	Review, case reports, research proposal report or conference abstracts

### Data extraction

Data extraction from each selected study was conducted independently by two reviewers (CY and HZ), using a structured form and checked by the third reviewer (KP-B). The extraction form mainly includes information of year of publication, the number of patients evaluated, study population characteristics, parameters of the intervention, pain ratings, and NCV data before and after NINM intervention. We used WebPlotDigitizer v.3.11^77^ to extract data from relevant graphs. The extracted data were tabulated, coded, and then imported into a dataset for analysis.

### Risk of bias assessment

The risk of bias of the selected studies was evaluated by two reviewers (KP-B and HZ) using the revised Cochrane risk-of-bias tool, RoB 2.0, for RCTs^78^ and MINORS score for Quasi-experiments^79^. The RoB 2.0 tool evaluates risk of bias of RCTs in five domains: (a) bias arising from the randomization process; (b) bias due to deviations from intended interventions; (c) bias due to missing outcome data; (d) bias in measurement of the outcome; (e) bias in selection of the reported result. We classified the studies in a low, high, and unclear risk of bias based on Cochrane handbook recommendations^80^. We used the MINORS tool^79^ to assess the risk of bias of QE studies. This tool considered three possible scores for each item from 0 to 2; 0 for not reported information, 1 for the information reported inadequately, and 2 for well-reported information. We considered for the overall risk of bias assessment that high risk of bias is indicated by scores less than 16 points, and low risk of bias indicated by 16 to 24 points, similar to previous studies^81,82^. Any disagreement was solved through discussion between the reviewers (KP-B and HZ). If a full consensus could not be reached between the two reviewers after an exhaustive discussion, the opinion of a third reviewer (FF) was obtained, and the proceeding majority consensus was taken. The publication bias across the RCTs was evaluated visually and statistically by the funnel plot and the Egger’s test, respectively.

### Data synthesis

We presented results separately based on study design (RCTs vs. QE), given the differences in the quality of evidence between these two types of studies.

Then, with the extracted data, we performed an exploratory meta-analysis as for our primary outcomes (pain intensity rating scales and NCV measurements). Although within the treatment categories are interventions with a potential different mechanism of action and stimulation parameters, we decided to do an exploratory synthesis to compare across the spectrum of the available non-invasive neuromodulation techniques. When possible, we used pre and post scores of the pain analog scales for each pain-related outcome and NCV data to calculate the mean difference between groups. The difference was then converted to an effect size (ES). Given that Cohen’s d has a slight bias to overestimate in small sample sizes, we adjusted Cohen’s d to Hedge’s g by applying a correction factor.

The pain and NCV outcomes (motor and sensory) were analyzed according to the following subgroups, if we got sufficient data: (a) type of the stimulation (central versus peripheral, electrical versus magnetic); (b) number of intervention sessions (less than 20 sessions and more than 20 sessions); (c) frequency of stimulation ( less than 3 times a week and ≥ times a week; (d) control group type (sham versus no-sham control intervention); (e) patient's response to analgesics (resistant or intolerant to analgesic medications due to severe side effects, not resistant to analgesic medications, mixed or not mentioned); and (f) two timepoints (immediately after stimulation and more than 1-month after stimulation).

In addition, we assessed heterogeneity using I^2^ statistic considering low heterogeneity when I^2^ < 40%^80^. We consider the random-effects models appropriate to use due to the overall heterogeneity evaluation (in population and intervention)^83^. Meta-regression was used to examine the impact of potential moderators and confounders (number of sessions, pain score at baseline, and predefined subgroups) on study effect size. And publication bias was assessed unless the number of studies pooled for each meta-analysis was less than ten^84^. The data was processed using Stata v15.0 software (StataCorp LLC).

### Human participants statement

The included studies were approved by the Regional Ethical Review Board of the corresponding country (United States of America and China) and followed the guidelines according to the Declaration of Helsinki as well as Good Clinical Practice. The included participants received both verbal and written information about the study and gave their written informed consent.

## Supplementary information

Supplementary Information

## Data Availability

The datasets generated during and/or analyzed during the current study are available from the corresponding author on reasonable request.
