# Supplementary material for: Non-invasive neuromodulation effects on painful diabetic peripheral neuropathy: a systematic review and meta-analysis
Source: Sci Rep. 2020 Nov 5;10:19184. doi: 10.1038/s41598-020-75922-9 (PMC7645738; doi:10.1038/s41598-020-75922-9)

**Non-Invasive Neuromodulation Effects on Painful Diabetic Peripheral Neuropathy:** **A systematic review and meta-analysis**

**Authors**

Huiyan Zeng^1,#^, Kevin Pacheco-Barrios^2,3,#^, Ying Cao^4^, Ying Li^4^, Jinming Zhang^1^, Caifeng Yang^1*^, Felipe Fregni^2*^

^1^Department of Endocrinology, The Second Affiliated Hospital of Guangzhou University of Chinese Medicine, Guangzhou, Guangdong, China.

^2^Neuromodulation Center and Center for Clinical Research Learning, Spaulding Rehabilitation Hospital and Massachusetts General Hospital, Harvard Medical School, Boston, USA.

^3^Universidad San Ignacio de Loyola, Vicerrectorado de Investigación, Unidad de Investigación para la Generación y Síntesis de Evidencias en Salud. Lima, Peru.

^4^Department of Endocrinology, Nanfang Hospital, Southern Medical University, Guangzhou, Guangdong, China.

# First authors

* Co-corresponding author: Felipe Fregni and Caifeng Yang contributed equally to this article.

**Corresponding:**

**Felipe Fregni**

Neuromodulation Center and Center for Clinical Research Learning, Spaulding Rehabilitation Hospital and Massachusetts General Hospital, Harvard Medical School, Boston, USA.

Address: 96 13th Street, Charlestown, Boston, MA, United States

Phone: 1 617 952 6153, Fax: 1 617 952 6150

Email: [Fregni.Felipe@mgh.harvard.edu](mailto:Fregni.Felipe@mgh.harvard.edu)

**Caifeng Yang**

Department of Endocrinology, The Second Affiliated Hospital of Guangzhou University of Chinese Medicine,Guangzhou, Guangdong, China

Address: 261 Datong Road, Er Sha Island, Guangzhou,510105, China.

Phone: 86 135 3941 6304,

Email: yuzhuyou@163.com

**Supplementary materials:**

**Supplementary material 1: Prism checklist.**

| **Section/Topic** | | **#** |  | **Checklist item** | **Reported on page #** | | |  | |  |
| --- | --- | --- | --- | --- | --- | --- | --- | --- | --- | --- |
|  | |  |  |  |  | | |  | |  |
|  | |  |  |  |  | | |  | |  |
| **TITLE** | |  |  |  |  | | |  | |  |
| Title | | 1 |  | Identify the report as a systematic review, meta-analysis, or | 01 | | |  | |  |
|  | |  |  | both. |  |  |  |  | |  |
|  | |  |  |  |  | | |  | |  |
|  | |  |  |  |  | | |  | |  |
|  | |  |  |  |  | | |  | |  |
| **ABSTRACT** | |  |  |  |  | | |  | |  |
| Structured | | 2 |  | Provide a structured summary including, as applicable: |  | | |  | |  |
| summary | |  |  | background; objectives; data sources; study eligibility |  | | |  | |  |
|  | |  |  | criteria, participants, and interventions; study appraisal and | 03 | | |  | |  |
|  | |  |  | synthesis methods; results; limitations; conclusions and |  |  |  |  | |  |
|  | |  |  |  |  | | |  | |  |
|  | |  |  | implications of key findings; systematic review registration |  | | |  | |  |
|  | |  |  | number. |  | | |  | |  |
|  | |  |  |  |  | | |  | |  |
|  | |  |  |  |  | | |  | |  |
| **INTRODUCTION** | | |  |  |  | | |  | |  |
| Rationale | | 3 |  | Describe the rationale for the review in the context of what | 05 | | |  | |  |
|  | |  |  | is already known. |  |  |  |  | |  |
|  | |  |  |  |  | | |  | |  |
|  | |  |  |  |  | | |  | |  |
| Objectives | | 4 |  | Provide an explicit statement of questions being addressed |  | | |  | |  |
|  | |  |  | with reference to participants, interventions, comparisons, | 06 | | |  | |  |
|  | |  |  | outcomes, and study design (PICOS). |  | | |  | |  |
|  | |  |  |  |  | | |  | |  |
|  | |  |  |  |  | | |  | |  |
| **METHODS** | |  |  |  |  | | |  | |  |
| Protocol and | | 5 |  | Indicate if a review protocol exists, if and where it can be |  | | |  | |  |
| registration | |  |  | accessed (e.g., Web address), and, if available, provide | 06 | | |  | |  |
|  | |  |  | registration information including registration number. |  | | |  | |  |
|  | |  |  |  |  | | |  | |  |
| Eligibility | | 6 |  | Specify study characteristics (e.g., PICOS, length of follow- |  | | |  | |  |
| criteria | |  |  | up) and report characteristics (e.g., years considered, | 07 | | |  | |  |
|  | |  |  | language, publication status) used as criteria for eligibility, |  |  |  |  | |  |
|  | |  |  |  |  | | |  | |  |
|  | |  |  | giving rationale. |  | | |  | |  |
|  | |  |  |  |  | | |  | |  |
| Information | | 7 |  | Describe all information sources (e.g., databases with |  | | |  | |  |
| sources | |  |  | dates of coverage, contact with study authors to identify | 06 | | |  | |  |
|  | |  |  | additional studies) in the search and date last searched. |  | | |  | |  |
|  | |  |  |  |  | | |  | |  |
| Search | | 8 |  | Present full electronic search strategy for at least one |  | | |  | |  |
|  | |  |  | database, including any limits used, such that it could be | 06 | | |  | |  |
|  | |  |  | repeated. |  | | |  | |  |
|  | |  |  |  |  | | |  | |  |
| Study | | 9 |  | State the process for selecting studies (i.e., screening, |  | | |  | |  |
| selection | |  |  | eligibility, included in systematic review, and, if applicable, | 06-07 | | |  | |  |
|  | |  |  | included in the meta-analysis). |  | | |  | |  |
|  | |  |  |  |  | | |  | |  |
| Data | | 10 |  | Describe method of data extraction from reports (e.g., |  | | |  | |  |
| collection | |  |  | piloted forms, independently, in duplicate) and any | 07 | | |  | |  |
| process | |  |  | processes for obtaining and confirming data from |  |  |  |  | |  |
|  |  |  |  |  |  | | |  | |  |
|  | |  |  | investigators. |  | | |  | |  |
|  | |  |  |  |  | | | | |  |
|  | Data items | 11 | List and define all variables for which data were sought | | |  |  | |  | |
|  |  |  | (e.g., PICOS, funding sources) and any assumptions and | | | 08 | | |  | |
|  |  |  | simplifications made. | | |  |  | |  | |
|  |  |  |  | | |  |  | |  | |
|  | Risk of bias in | 12 | Describe methods used for assessing risk of bias of | | |  |  | |  | |
|  | individual |  | individual studies (including specification of whether this | | | 07-08 | | |  | |
|  | studies |  | was done at the study or outcome level), and how this | | |  |  |  |  | |
|  |  |  |  |  |  |  |  | |  | |
|  |  |  | information is to be used in any data synthesis. | | |  |  | |  | |
|  |  |  |  | | |  |  | |  | |
|  | Summary | 13 | State the principal summary measures (e.g., risk ratio, | | | NA | | |  | |
|  | measures |  | difference in means). | | |  |  |  |  | |
|  |  |  |  |  |  |  |  | |  | |
|  |  |  |  | | |  |  | |  | |
|  | Synthesis of | 14 | Describe the methods of handling data and combining | | |  |  | |  | |
|  | results |  | results of studies, if done, including measures of | | |  | 08 | | | |
|  |  |  | consistency (e.g., I^2^) for each meta-analysis. | | |  |  | |  | |
|  |  |  |  | | |  |  | |  | |
|  | Risk of bias | 15 | Specify any assessment of risk of bias that may affect the | | |  |  | |  | |
|  | across studies |  | cumulative evidence (e.g., publication bias, selective | | |  | NA | | | |
|  |  |  | reporting within studies). | | |  |  | |  | |
|  |  |  |  | | |  |  | |  | |
|  | Additional | 16 | Describe methods of additional analyses (e.g., sensitivity | | |  |  | |  | |
|  | analyses |  | or subgroup analyses, meta-regression), if done, indicating | | |  | 08-09 | | | |
|  |  |  | which were pre-specified. | | |  |  | |  | |
|  |  |  |  | | |  |  | |  | |
|  |  |  |  | | |  |  | |  | |
|  | **RESULTS** |  |  | | |  |  | |  | |
|  | Study | 17 | Give numbers of studies screened, assessed for eligibility, | | |  |  | |  | |
|  | selection |  | and included in the review, with reasons for exclusions at | | | 09 | | |  | |
|  |  |  | each stage, ideally with a flow diagram. | | |  |  | |  | |
|  |  |  |  | | |  |  | |  | |
|  | Study | 18 | For each study, present characteristics for which data were | | |  |  | |  | |
|  | characteristics |  | extracted (e.g., study size, PICOS, follow-up period) and | | | 10-14 | | |  | |
|  |  |  | provide the citations. | | |  |  | |  | |
|  |  |  |  | | |  |  | |  | |
|  | Risk of bias | 19 | Present data on risk of bias of each study and, if available, | | | 15-17 | | |  | |
|  | within studies |  | any outcome level assessment (see item 12). | | |  |  |  |  | |
|  |  |  |  |  |  |  |  | |  | |
|  |  |  |  | | |  |  | |  | |
|  | Results of | 20 | For all outcomes considered (benefits or harms), present, | | |  |  | |  | |
|  | individual |  | for each study: (a) simple summary data for each | | | 17 | | |  | |
|  | studies |  | intervention group (b) effect estimates and confidence | | |  |  |  |  | |
|  |  |  |  |  |  |  |  | |  | |
|  |  |  | intervals, ideally with a forest plot. | | |  |  | |  | |
|  |  |  |  | | |  |  | |  | |
|  | Synthesis of | 21 | Present results of each meta-analysis done, including | | | 17-18 | | |  | |
|  | results |  | confidence intervals and measures of consistency. | | |  |  |  |  | |
|  |  |  |  |  |  |  |  | |  | |
|  |  |  |  | | |  |  | |  | |
|  | Risk of bias | 22 | Present results of any assessment of risk of bias across | | |  | 17 | | | |
|  | across studies |  | studies (see Item 15). | | |  |  |  |  |  |
|  |  |  |  |  |  |  |  | |  | |
|  |  |  |  | | |  |  | |  | |
|  | Additional | 23 | Give results of additional analyses, if done (e.g., sensitivity | | |  | 18-19 | | | |
|  | analysis |  | or subgroup analyses, meta-regression [see Item 16]). | | |  |  |  |  |  |
|  |  |  |  |  |  |  |  | |  | |
|  |  |  |  | | |  |  | |  | |
|  |  |  |  | | |  |  | |  | |
|  | **DISCUSSION** |  |  | | |  |  | |  | |
|  | Summary of | 24 | Summarize the main findings including the strength of | | |  |  | |  | |
|  | evidence |  | evidence for each main outcome; consider their relevance | | | 19-20 | | |  | |
|  |  |  | to key groups (e.g., healthcare providers, users, and policy | | |  |  |  |  | |
|  |  |  |  |  |  |  |  | |  | |
|  |  |  | makers). | | |  |  | |  | |
|  |  |  |  | | |  |  | |  | |
|  | Limitations | 25 | Discuss limitations at study and outcome level (e.g., risk of | | | 22 | | |  | |
|  |  |  | bias), and at review-level (e.g., incomplete retrieval of | | |  |  |  |  | |
|  |  |  |  |  |  |  |  | |  | |
|  |  |  |  | | |  |  | |  | |
|  |  |  | identified research, reporting bias). | | |  | | | | |
|  |  |  |  | | |  | | | | |
|  | Conclusions | 26 | Provide a general interpretation of the results in the | | |  | | | | |
|  |  |  | context of other evidence, and implications for future | | | 22-23 | | | | |
|  |  |  | research. | | |  | | | | |
|  |  |  |  | | |  | | | | |
|  |  |  |  | | |  | | | | |
|  | **FUNDING** |  |  | | |  | | | | |
|  | Funding | 27 | Describe sources of funding for the systematic review and | | |  | | | | |
|  |  |  | other support (e.g., supply of data); role of funders for the | | | 02 | | | | |
|  |  |  | systematic review. | | |  | | | | |
|  |  |  |  | | |  | | | | |
|  |  |  |  | | |  | | | | |

Reference: Moher D, Liberati A, Tetzlaff J, Altman DG, The PRISMA Group (2009). Preferred Reporting Items for Systematic Reviews and Meta-Analyses: The PRISMA Statement. PLoS Med 6(7): e1000097. doi:10.1371/journal.pmed1000097.

**Supplementary material 2: Search strategy.**

| **Database** | **Date** | **Terms** |
| --- | --- | --- |
| **MEDLINE**  PubMed | **2019-8-3** | **#1：NON-INVASIVE STIMULATION**  ("noninvasive brain stimulation"[TIAB] OR "non-invasive brain stimulation"[TIAB] OR "neuromodulation"[TIAB] OR "NIBS"[TIAB] OR "motor cortex stimulation"[TIAB]) OR "transcranial magnetic stimulation"[Mesh] OR "transcranial magnetic stimulation"[TIAB] OR "TMS"[TIAB] OR "rTMS"[TIAB] OR “Transcranial Electric Stimulation”[TIAB] OR "transcranial direct current stimulation"[Mesh] OR "transcranial direct current stimulation"[TIAB] OR "tDCS"[TIAB]OR "transcranial Alternating Current Stimulation"[TIAB] OR “tACS”[TIAB] OR "transcranial Random Noise Stimulation"[TIAB] OR “tRNS”[TIAB] OR  "Transcutaneous Electric Nerve Stimulation"[Mesh] OR "Transcutaneous Electric Nerve Stimulation"[TIAB] OR "Transcutaneous Electrical Nerve Stimulation"[TIAB] OR “TENS“[TIAB] OR "transcutaneous vagus nerve stimulation"[Mesh] OR "tVNS"[TIAB] OR " transcranial ultrasound stimulation"[Mesh] OR "TUS"[TIAB]  **#2：Diabetes**  ("diabetes"[Mesh] OR "diabetic"[Mesh] OR "Diabetes Mellitus"[Mesh] OR " DM"[Mesh] OR " diabetes "[TIAB] OR " diabetic "[TIAB] OR " Diabetes Mellitus "[TIAB] OR " DM"[TIAB])  #1 AND #2 |
| Web of science | **2019-8-2** | #1 NON-INVASIVE STIMULATION  **TS=(**'transcranial direct current stimulation' OR 'noninvasive brain stimulation' OR 'transcranial direct current stimulation' OR 'neuromodulation'OR 'nibs' OR 'tdcs' OR 'transcranial magnetic stimulation' OR 'tms' OR 'rtms' OR 'motor cortex stimulation' OR 'transcutaneous electric nerve stimulation' OR 'tens' OR 'transcutaneous vagus nerve stimulation' OR 'tVNS' OR ' transcranial ultrasound nerve stimulation' OR 'TUS')  #2 diabetes  **TS=(**'diabetes' OR ' diabetic' OR ' diabetes mellitus' OR 'DM' )  #1 AND #2 |
| **EMBASE** | **2019-8-2** | ('transcranial direct current stimulation' OR 'noninvasive brain stimulation' OR 'non-invasive brain stimulation' OR 'transcranial direct current stimulation' OR 'neuromodulation' OR 'nibs' OR 'tdcs' OR 'transcranial magnetic stimulation'  OR 'tms' OR 'rtms' OR 'motor cortex stimulation' OR 'transcutaneous electric nerve stimulation' OR 'tens' OR 'transcutaneous vagus nerve stimulation' OR 'tVNS' OR 'transcranial ultrasound nerve stimulation' OR 'TUS') AND ('diabetic peripheral neuropathy' OR 'DPN' OR 'DPND' OR 'PND' OR 'peripheral nerve' OR 'peripheral neuropathy' OR 'peripheral nerve diseases' OR 'polyneuropathy') AND 'article'/it |
| **Scopus** | **2019-8-4** | TITLE-ABS-KEY (“transcranial direct current stimulation” OR “noninvasive brain stimulation” OR “non-invasive brain stimulation” OR  “transcranial direct current stimulation” OR “neuromodulation” OR  “nibs" OR  “tdcs” OR  “transcranial magnetic stimulation” OR  “tms” OR  “rtms" OR  “motor cortex stimulation” OR  “transcutaneous electric nerve stimulation” OR  “transcutaneous electrical nerve stimulation” OR  “tens” OR  “transcutaneous vagus nerve stimulation” OR “tVNS” OR  “transcranial ultrasound nerve stimulation” OR “TUS”) AND TITLE-ABS-KEY(“diabetic peripheral neuropathy” OR “DPN” OR “peripheral nerve” OR “peripheral neuropathy” OR “peripheral nerve diseases” OR “PND” OR “DPND” OR “polyneuropathy”) |
| **LILACS** | **2019-8-12** | #1  (tw:(“transcranial direct current stimulation”)) OR (tw:(“noninvasive brain stimulation”)) OR (tw:(“transcranial direct current stimulation”)) OR (tw:( neuromodulation)) OR (tw:(“nibs”)) OR (tw:(“tdcs”)) OR (tw:(“transcranial magnetic stimulation”)) OR (tw:(“tms”)) OR (tw:(“rtms”)) OR (tw:(“motor cortex stimulation”)) OR (tw:(“transcutaneous electric nerve stimulation”)) OR (tw:(“tens”)) OR (tw:(“transcutaneous vagus nerve stimulation”)) OR (tw:(“tVNS”)) OR (tw:(“transcranial ultrasound nerve stimulation”)) OR (tw:(“TUS”))  #2  (tw:(“diabetes”)) OR (tw:( diabetic)) OR (tw:(diabetes mellitus)) OR (tw:(DM))  #1 AND #2 |
| **CNKI** | **2019-9-19** | #1  "非侵入性脑刺激"[主题] OR "非侵入性神经调节"[主题] OR "经颅磁刺激" [主题] OR "经颅电刺激"[主题] OR "经颅直流电刺激"[主题] OR " 经颅交流电刺激"[主题] OR "经颅随机噪声刺激"[主题] "经皮神经电刺激"[主题] OR "经皮迷走神经刺激"[主题] OR "经颅超声神经刺激"[主题]  ("noninvasive brain stimulation"[Subject term] OR "neuromodulation"[Subject term] OR "transcranial magnetic stimulation" [Subject term] OR "transcranial direct current stimulation"[Subject term] OR "transcranial direct current stimulation"[Subject term] OR "transcranial Alternating Current Stimulation"[Subject term] OR "transcranial Random Noise Stimulation"[Subject term] "Transcutaneous Electric Nerve Stimulation"[Subject term] OR " transcutaneous vagus nerve stimulation "[Subject term] OR "transcranial ultrasound nerve stimulation"[Subject term])  #2  “糖尿病周围神经病变” [Subject term] OR “周围神经病变” [Subject term]  (“Diabetic peripheral neuropathy” [Subject term] OR “peripheral neuropathy” [Subject term])  #1 AND #2 |

**Supplementary material 3: studies that were evaluated in full-text and were excluded.**

| **N** | **Author** | **Year** | **Title** | **Reason for exclusion** |
| --- | --- | --- | --- | --- |
| 1 | Cauda, F.  et al | 2009 | Altered resting state in diabetic neuropathic pain | Lack of pain score or nerve conduction velocity data |
| 2 | Gokcay, I.  et al | 2015 | Striking efficacy of transcutaneous electrical nerve stimulations in peripheral polyneuropathies in chronic kidney disease, diabetes mellitus and primary hypertension | Conference abstract |
| 3 | Julka, I. S. et al | 1998 | Beneficial effects of electrical stimulation on neuropathic symptoms in diabetes patients | Retrospective study |
| 4 | Kavak, S.  et al | 2010 | Effects of High-Rate Frequency Modulation Treatment on Malondialdehyde in Diabetic Polyneuropathy | Lack of pain score or nerve conduction velocity data |
| 5 | Kavak, S. et al | 2010 | Effects of transcutaneous electrical nerve stimulation on motor and sensorial nerves for diabetic polyneuropathy patients by use of electromyography | Lack of pain score or nerve conduction velocity data |
| 6 | Bosi, A.  et al | 2008 | High-Tone External Muscle Stimulation in End-Stage Renal Disease: Effects on Symptomatic Diabetic and Uremic Peripheral Neuropathy | Not all patients were DPN |
| 7 | Lee, S.  et al | 2013 | Electroacupuncture for treating painful diabetic neuropathy: Study protocol for a randomized, patient-assessor blinded, controlled pilot clinical trial | Conference abstract |
| 8 | Lee, S. et al | 2013 | Electroacupuncture to treat painful diabetic neuropathy: Study protocol for a three-armed, randomized, controlled pilot trial | Research proposal report |
| 9 | Maruszewska, A.  et al | 2016 | The Use of Combination Therapy TENS and Alpha-Lipoic Acid in Treatment of Diabetic Polyneuropathy of the Lower Limbs | Combined with other treatments |
| 10 | Onesti, E. et al | 2012 | The effect of H-coil repetitive transcranial magnetic stimulation on painful diabetic neuropathy: A randomized placebo-controlled crossover study | Conference abstract |
| 11 | Pourmomeny, A. A. et al | 2009 | The effect of electroanalgsia on pain relief in patient with diabetic neuropathy type II | Lack of pain score or nerve conduction velocity data |
| 12 | Saadat, Z. et al | 2017 | Dose postural control improve following application of transcutaneous electrical nerve stimulation in diabetic peripheral neuropathic patients? A randomized placebo control trial | Lack of pain score or nerve conduction velocity data |
| 13 | Sagliker, Y. et al | 2017 | Transcutaneous electrical nerve stimulation (TENS) in peripheral polyneuropathy in diabetes mellitus, chronic kidney disease and primary hypertension | Conference abstract |
| 14 | Satla, M. S. et al | 2019 | Efficacy of percutaneous electrical neurostimulation in the management of diabetic peripheral neuropathic pain | Conference abstract |
| 15 | Tartaglia, G.et al | 2011 | Pain relief by deep repetitive transcranial magnetic stimulation applied with the H-coil | Conference abstract |
| 16 | Turgut, N. et al | 2009 | Cortical disinhibition in diabetic patients with neuropathic pain | Lack of pain score or nerve conduction velocity data |
| 17 | Upton, G. A. et al. | 2017 | The influence of transcutaneous electrical nerve stimulation parameters on the level of pain perceived by participants with painful diabetic neuropathy: A crossover study. | Lack of pain score or nerve conduction velocity data |
| 18 | Jannu, C. et al | 2018 | Efficacy of Interferential Therapy Versus Transcutaneous Electrical Nerve Stimulation to Reduce Pain in Patients with Diabetic Neuropathy | No appropriate control group |
| 19 | Reichstein, L. et al. | 2005 | Effective treatment of symptomatic diabetic polyneuropathy by high-frequency external muscle stimulation | No appropriate control group |
| 20 | Wang, H. | 2013 | Curative effect of transcutaneous electric nerve stimulation combined with microwave on painful diabetic peripheral neuropathy | Combined with other treatments |
| 21 | Humpert. PM, et al. | 2009 | External Electric Muscle Stimulation Improves Burning  Sensations and Sleeping Disturbances in Patients with Type 2  Diabetes and Symptomatic Neuropathy | No appropriate control group |
| 22 | A. V. Musaev, et al. | 2003 | The Use of Pulsed Electromagnetic Fields with Complex Modulation in the Treatment of Patients with  Diabetic Polyneuropathy | No appropriate control group |
| 23 | Michael I. Weintraub, et al. | 2004 | Pulsed Magnetic Field Therapy in Refractory Neuropathic Pain Secondary to Peripheral Neuropathy: Electrodiagnostic Parameters—Pilot Study | Not all patients were DPN |

**Supplementary material 4: Included studies characteristics**

| **Study** | **Study Design** | **Participants that completed the study (n, age, years, mean ± SD, and sex)** | **Diagnosis of DPN** | **Stable Analgesic Medication Treatment** | **Intervention(duration and frequency of sessions)** | **Outcomes for neuropathy Measurement** | **Assessment timing** | **Conclusion** |
| --- | --- | --- | --- | --- | --- | --- | --- | --- |
| Onesti et al. 2013 | Double Crossover | Real-sham rTMS group: n = 11 Age=70.7±9.5 Sex=4 female, 7 male.  Sham-real rTMS group: n = 12 Age=70.7±9.5 Sex=5 female, 7 male; | DN4 score ≥4 | YES | Active rTMS: 30 consecutive trains of 50 stimuli delivered by a H-coil targeting Cz area, with 20 Hz, at 100% of resting motor Th (RMT), separated by intertrain intervals lasting 30 s. 20 min each time for 5 consecutive days.  Sham rTMS: using a sham coil with the same parameters. 20 min each time for 5 consecutive days. | VAS(0-100)  RIII reflex(the size of the RIII reflex and RIII threshold) | Before, immediately after and 3 weeks after the treatment | Deep H-coil rTMS provides pain relief in patients with diabetic neuropathy. |
| Weintraub et al. 2009 | RCT | PEMF Group: n = 90 Age= 61.1±10.4 Sex=51 female, 39 male.  Sham Group: n = 104 Age= 60.6±12.4 Sex=58 female, 46 male; | Dyck stage II or III | YES | PEMF Group: 25 times a second at maximum 1500 revolutions per minute of pulsed electromagnetic stimulation. A maximum of 2 hours a day in divided sessions of 10 to 30 minutes for 3 months.  Sham Group: sham exposure with the same sessions. | VAS (0–10)  NPS(0-100)  PGIC | Before, immediately after the treatment | PEMF was noneffective in reducing neuropathic pain |
| Serry et al. 2016 | RCT | TENS group: n = 20 Age=51.6±4.75 Sex=12female, 8 male.  Exercise group: n = 20 Age=51.7±4.44 Sex=10female, 10 male.  Pharmacological group: n = 20 Age=51.95±4.38 Sex=10 female, 10 male; | No data provided | YES | TENS group: received TENS (with a low frequency of 15 Hz and a pulse width of 250 µs) on both lower limbs, three times per week for 8 weeks, in addition to their regular pharmacological therapy.  Exercise group: received aerobic exercise on a stationary bicycle, three times per week for 8 weeks, in addition to their regular pharmacological therapy.  Pharmacological group: received only their regular pharmacological therapy for peripheral neuropathy (nerve growth stimulant; vitamin B complex) and oral hypoglycemic drugs or insulin. | VAS  Sensory NCS | Baseline, immediately after the treatment. | TENS is better than exercise training program in relieving pain in patients with DPN.  In contrast, neither TENS nor exercise showed any significant eﬀect on medial plantar CV in patients with DPN |
| Oyibo et al. 2004 | Crossover | n = 14 Age= 57.7±10.7  No other demographic characteristics data provided | NDS > 4/10 | NO | Active treatment group: wear a pair of knitted silver-plated nylon-Dacron™ stocking electrodes (delivered a subsensory dose of 50 V of pulsed direct current) for 8 h a night, every night for a 6-week.  Control group: wear a similar stocking electrode and microstimulator units, delivering an insignificant current(5 V), with the same sessions. | VAS | Baseline, immediately after the treatment. | No evidence shows that electrical stimulation therapy through stocking electrodes is more efficacious than stockings alone in the treatment of painful diabetic neuropathy |
| Naderi et al. 2015 | RCT | PRF group: n = 30 Age= 56.76 ± 6.94  TENS group: n = 30 Age= 56.63 ± 5.86  Sex（total）：29 female, 31 male; | NRS≥4 | YES | PRF group: receive PRF lumbar sympathectomy（L4-L5 level） in the operating room  TENS group: received TENS (80 Hz, 50 Amp, 0.2 ms square pulses, 2 to 3 times sensory threshold) for 20 minutes from a TENS stimulator (E3 model, Omron, [Omron location]). Ten TENS sessions were performed every other day. | NRS | Baseline, 1 week, 1month, and 3 months after the treatment | Both PRF sympathectomy and TENS can reduce lower extremity pain in patients with PDPN. However, PRF sympathectomy seems to be more eﬀective than TENS |
| Kumar et al. 1997 | RCT | Electrotherapy group: n = 18 Age= 53+4 Sex=11 female, 7 male  Sham group: n = 13 Age= 59+3 Sex=8 female, 5 male; | Pain Scores≥2 | NO | Electrotherapy group: treated each lower extremity for 30 min daily for 4 weeks at home.  Sham group: using a sham device (inactive electrodes) with the same parameters and sessions | Pain grade | Baseline, immediately after and 1month after the treatment | Transcutaneous electrotherapy reduced the pain and discomfort of peripheral neuropathy in 15 of the 18 (83%) patients. |
| Kim et al. 2013 | RCT | M1 group: n = 20 Age=59.60±13.15 Sex=11 female, 9 male.  DLPFC group: n = 20 Age= 61.60±10.27 Sex=12 female, 8 male.  Sham group: n = 20 Age= 63.50±8.75 Sex=12 female, 8 male; | NTSS>6 | YES | M1 group: The anode (saline-soaked electrodes, 5×5cm) was placed over C3 (EEG 10/20 system) and the cathode over the contralateral supraorbital area. A constant current with an intensity of 2 mA was used for a single 20-minute session for 5 consecutive days  DLPFC group: the anode was placed over F3 (EEG 10/20 system) and the cathode over the contralateral supraorbital area. A constant current with an intensity of 2 mA was used for a single 20-minute session for 5 consecutive days。  Sham group: the same electrode positions were used as in anodal M1 stimulation, but the stimulator was on for only 30 seconds | VAS  CGI score  Anxiety score.  sleep quality  BDI  PPT | Baseline, immediately after, 2weeks and 4 weeks after the treatment | Five daily sessions of anodal tDCS over the M1, but not sham tDCS or anodal tDCS over the DLPFC, effectively reduce pain and increase PPT in patients with painful DPN |
| Hamza et al. 2000 | Crossover study | PENS group: n = 25 Age= 54±9  Sham group: n = 25 Age= 56±8  Sex（total）=28 female, 22 male; | NOT mentioned | YES | PENS group: received stipulated 30 min of active electrical stimulation treatment three times a week for 3 consecutive weeks.  Sham group: sham stimulation. | VAS  SF-36  BDI  POMS | VAS: baseline, before each treatment session, after each week of treatment, at the end of the 3-week treatment period with each modality  SF-36,BDI and POMS: 24 h before the first treatment and were repeated 48 h after completing the 3-week treatment session with each modality | PENS therapy produces short-term pain relief; improves mood, functionality, quality of sleep and decreases the oral nonopioid analgesic requirements in patients with painful DPN |
| Gossrau et al. 2011 | RCT | TENS group : n = 21 Age= 67.91±12.13  Placebo group: n = 19 Age= 65.95±7.05  No other demographic characteristics data provided | NRS≥4（10） | NO | TENS group: obtained a micro-TENS device [low-frequency (bursts of 2 Hz) microcurrent of 30–40 mA] treatment, the duration of the study was 4 weeks with three visits each week by individual appointment. Treatment sessions lasted 30 minutes each.  Placebo group: identical with the exception that the electrodes were not connected to the TENS device. | PDI  NPS  CES-D | Before and immediately after and 1 month after the treatment. | The pain reduction with the applied TENS is not superior to a placebo treatment |
| Lacigová et al. 2013 | cross-over study | MDM group: n = 30 Age= 62±7.2  Sham group: n = 30 Age= 62±7.2 | NOT mentioned | YES | MDM group: The treatment was comprised of 13 thirty-minute procedures using MDM electrotherapeutic device (ZAT a.s). The whole treatment lasted 10 days, twice a day for the first three days of treatment the modulation and then once per day.  Sham group: sham modulation. | VAS  TSS  BDI  SF-36 | One week before, daily within the 10-day exposure and 1 month after each treatment | The study did not demonstrate any positive effect of MDM on painful diabetic neuropathy compared to placebo, relative to pain or mental state evaluations |
| Kumar et al. 1998 | RCT | Electrotherapy group: n = 14 Age= 59+2 Sex=10 female, 4 male  Sham group: n = 9 Age= 58+4 Sex=3 female, 6 male; | pain grade >2 | YES | Electrotherapy group: treated by a portable unit (H-wave machine) that generated a biphasic exponentially decaying waveform (with pulse widths of 4 ms,≤35 mA, ≤35V and 2-70 Hz.) daily for 12 weeks.  Sham group: Sham stimulation | Pain grade | At the initiation of amitriptyline therapy, before and after the electrotherapy | Transcutaneous electrotherapy is effective in reducing the pain associated with peripheral neuropathy |
| Abdelkader et al. 2019 | Quasi-experiment | Insulin dependent group: n = 10 Age= 52.1± 6.45 Sex=6 female, 4 male  Non-insulin dependent group: n = 10 Age= 57.9 ± 10.22 Sex=5 female, 5 male; | Dyck severity score ≥ 2 | NO | All patients received five consecutive sessions of high-frequency rTMS | VAS | prior to and following the treatment | rTMS significantly reduced pain in both insulin dependent and non-insulin dependent patients with painful DPN. |
| Moharič et al. 2009 | RCT | TENS group: n = 46 Age= 62.4 ±6.5  Pregabalin group: n = 5 Age= 63.4 ±7.2  Combination group: n = 14 Age= 60.6 ±6.4  No other demographic characteristics data provided | MNSI≥2 | YES | TENS group: received TENS treatment at home with standard TENS parameters: rectangular, constant, monophasic impulse; frequency 100 Hz; impulse width 0.2 ms） for three weeks, three hours a day。  Pregabalin group: Pregabalin 2 × 75 mg/day during the first week and then 2 × 150 mg/day for two weeks, after that, it was reduced to 2 × 75 mg/day for one week, and then completely withdrawn.  Combination group: both agents were used in combination in the same way as in the first two groups | VAS  SF-36 | baseline, immediately after and 1 month after the treatment | TENS did not differ in efficacy from pregabalin and combined treatment and does not have side effects in DPN. |
| Bulut et al. 2011 | RCT | TENS group: n = 20 Age= 58.45 ± 15.9 Sex=12 female, 8 male.  Placebo TENS group: n = 20 Age=62.05 ± 19.9 Sex=11 female, 9 male; | ASA I-III | NO | TENS group: Electrostimulation with a frequency of 80 Hertz and an amplitude high enough to create paresthesia was used 30 minutes daily for 20 days.  Placebo TENS group: the electrodes were applied to the same region with a low amplitude 30 minutes daily for 20 days. | VAS  Pain grade | VAS; before and 5th, 10^th^ and 20th days of the procedure  Pain grade; before study and 20th day of the procedure. | TENS can be used as an efficient and safe treatment option especially in DPN patients in whom pharmacologic treatment is contraindicated or inefficient. |
| Yuanhong Ding et al. 2017 | RCT | TENS group: n = 30  Control group: n = 30  No demographic characteristics data provided | NOT mentioned | YES | TENS group: received TENS treatment(with frequency 70 ~ 100 Hz, pulse 0.1 ~ 0.3 ms), twice a day + Epalrestat 50mg,po.,tid, for 4 weeks.  Control group: Epalrestat 50mg, po., tid, for 4 weeks. | MCV  SCV  clinical effects | Before and immediately after treatment | TENS is safe and effective in the treatment of DPN. |
| Yonghong Guo et al. 2004 | RCT | Treatment group: n = 34 Age=61.06 ±1.76 Sex=12 female, 22 male.  Control group: n = 34 Age=56.41 ±2.37 Sex=17 female, 17 male; | NOT mentioned | YES | Treatment group: received transepidermal acupoint electric stimulation (HANS therapeutic instrument, density wave with a frequency of 50 Hz) 30 minutes per day for totally 2 courses of treatment divided by a 3-day interval. Each course contained 10 times treatment.  Control group: Mecobalamin 0.5mg, po., tid. The course of treatment is the same as the treatment group. | MCV  SCV  hemorheology | Before and immediately after treatment | Transepidermal acupoint electric stimulation obtained a better therapeutic effect in improving clinical symptoms and increasing conduction velocity of the injured nerves than Mecobalamin. |
| Wróbel et al. 2008 | RCT | Study group: n = 32 Age=53.6 ± 13.6 Sex=20 female, 12 male.  Control group: n = 29 Age=55.5 ± 10.4 Sex=16 female, 13 male; | simple clinical tests, including pinprick, temperature, and vibration perception (using a 128 Hz tuning fork), 10 g monoﬁlament pressure sensation at the distal halluces and ankle reﬂexes and was also conﬁrmed by electroneurography | YES | Study group: a low frequency PEMF of up to 100 μT with a complex sequence of pulses at a 180–195 Hz frequency electromagnetic waves and a 130 V/m electrical ﬁeld intensity for 15 days (three weeks, excluding Saturdays and Sundays).  Control group: sham stimulation | VAS  EuroQol EQ-5D  MOS Sleep Scale | Before and after each week treatment | Genuine magnetic ﬁeld exposure has no advantage over sham exposure in reducing pain intensity, improving quality of life, and decreasing sleep disturbances and HbA1c |
| Armstrong et al. 1997 | Quasi-experiment | n = 10  Age= 52.4± 10.2  Sex=3 female, 6 male | The University of Texas Diabetic Foot Classifi­cation System category II or lower. | NO | Electrical stimulation with a dose of 50 V of pulsed direct current (approximately 50 µA) at 100 pulses per sec. for 10 minutes, then 10 pulses per sec. for 10 minutes was delivered each hour over an 8-hr. period nightly for 1 month. | VAS | Before and immediately after and 1 month after treatment | Pulsed-dose electrical nerve stimulation appears to have promise as an analgesic for use in painful burning diabetic neuropathy |
| Bosi et al. 2005 | crossover | n = 31  Age: FREMS-placebo (sequence 1) = 63.1±3.1; Placebo-FREMS (sequence 2)= 59.2±3.1 | 1）SCV and/or MCV (<40 m/s in at least one nerve trunk of lower limbs);  2) vibration perception at big toe >25 V. | NO | FREMS: FREMS or placebo via four electrodes applied to the lower extremities for 30 min; Placebo consisted of no electric current transmission. Each treatment session was administered at intervals of at least 24 h, and each ten-session series lasted no more than 3 weeks. | VAS  MCV  SCV  SF36  VPT | Before and immediately after and 4 months after treatment | FREMS is a safe and effective therapy for neuropathic pain in patients with diabetes and is able to modify some parameters of periph-eral nerve function. |
| Forst et al. 2004 | RCT | TENS group: n=12 Age=57.6±11.5 Sex=6 female, 6 male.  Sham group: n=7 Age=59.4±8.6 Sex=3 female, 4 male. | NTSS： 4-16 | NO | TENS group: received TENS 4 Hz/280 ms; 5-70 mA,30 min per day, for 12 weeks.  Sham group: Sham TENS | NTSS-6  VAS | Baseline, after 6 weeks and 12weeks of treatment, | The new TENS device "Salutaris" is a convenient, non-pharmacological option for primary or adjuvant treatment of painful diabetic neuropathy |

DN4: Douleur Neuropathique 4 Questions; rTMS: repetitive transcranial magnetic stimulation; VAS: Visual analog scale; NPS: Neuropathy Pain Scale; PGIC: Patient’s Global Impression of Change; PI-NRS: Pain Intensity Numerical Rating Scale; PEMF: pulsed electromagnetic field therapy; TENS: transcutaneous electrical nerve Stimulation; HF: high-frequency external muscle Stimulation; NDS: neuropathy disability score; PRF: Pulsed Radio frequency Sympathectomy; tDCS: transcranial direct current stimulation; M1: primary motor cortex; DLPFC: dorsolateral prefrontal cortex; CGI: Clinical Global Impression; BDI: Beck Depression Inventory; PPT: pressure pain threshold; NTSS: neuropathy total symptom score; POMS: the Profile of Mood Status; SF-36: the MOS 36-Item Short-Form Health Survey; CES-D: Center for Epidemiologic Studies Depression Scale; IFT: Interferential therapy; MDM: “mesodiencephalic” modulation; MCV: motor conduction velocity; SCV: sensory conduction velocity; SEP: Somatosensory-evoked potential; PSP: Acupoint skin pain threshold; NIS LL: Neuropathy Impairment Score Low Limbs scale.

**Supplementary material 5: Subgroup meta-analysis**

(A) Frequency of the stimulation (<3 times a week; ≥3 times a week); (B) The type of control group (sham stimulation as a control or not); (C) Patient's response to analgesics (resistant or intolerant to analgesic medications due to severe side effects; not resistant to analgesic medications; and mixed groups or not mentioned); (D) Follow-up (more than 1-month after stimulation);(E) Type of peripheral NINMs(electric or electromagnetic); (F)Current frequency of peripheral electric NINMs(low(<10Hz), high(>10Hz) or mixed). All outcomes are shown with 95% confidence intervals.

(A) Frequency of stimulation


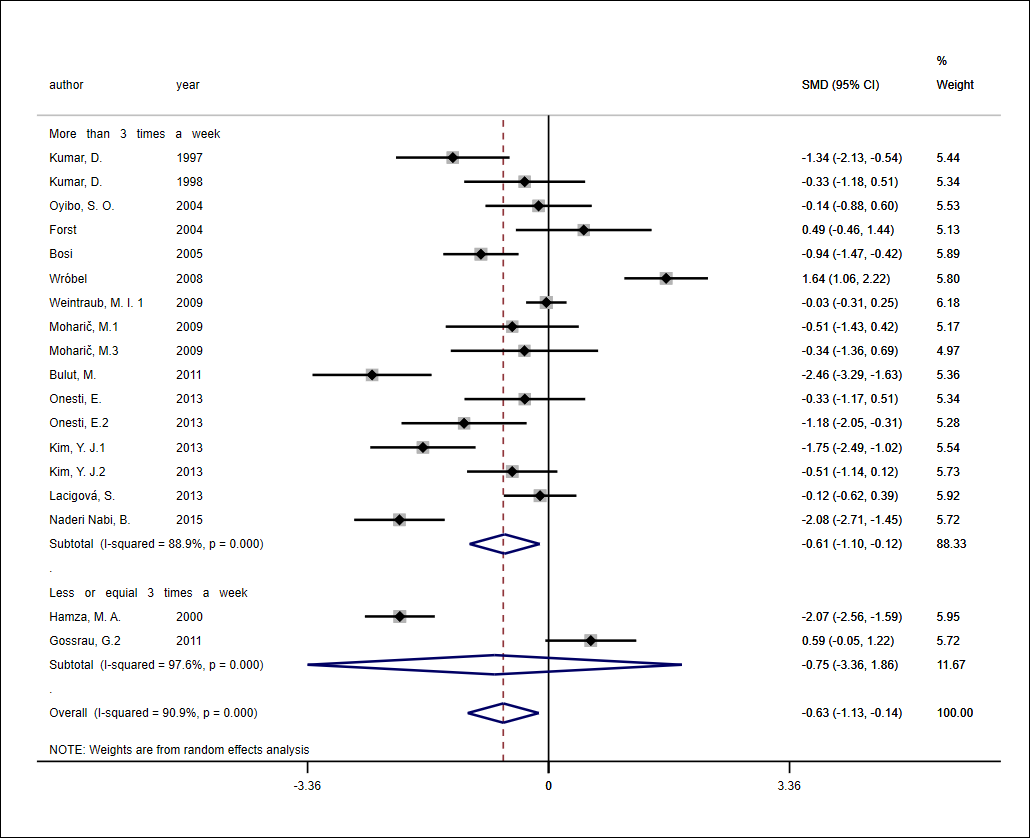


(B) Type of control group


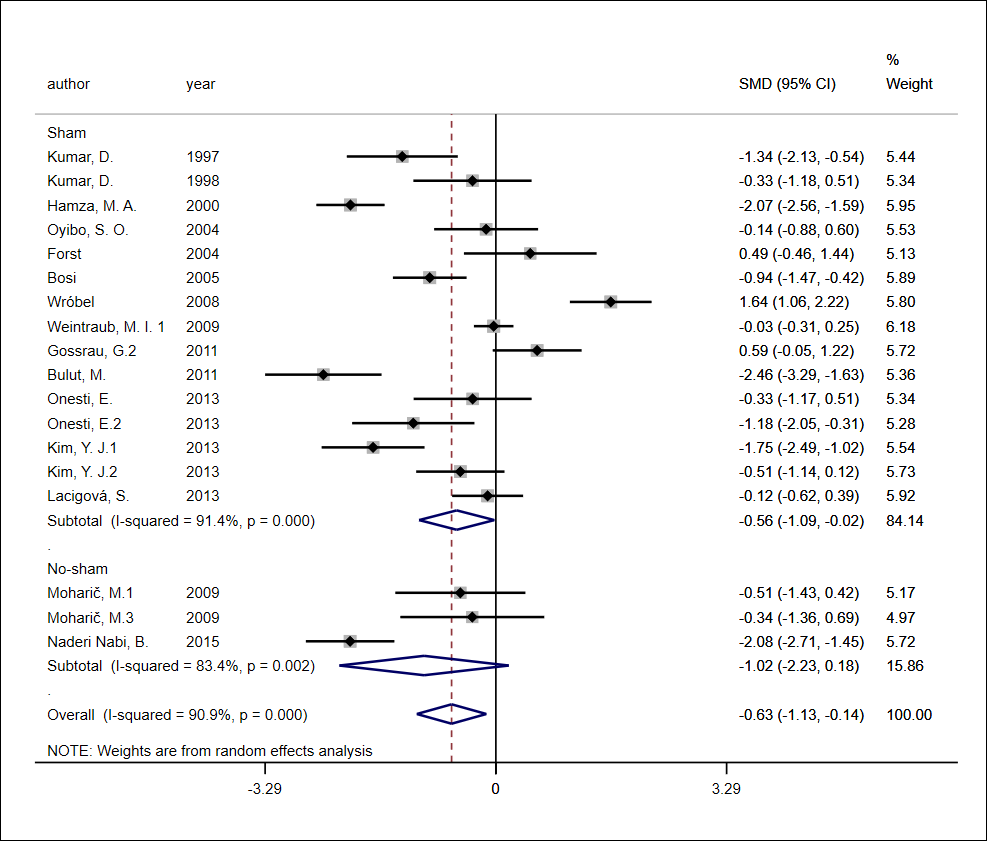


(C) Patient’s response to analgesics


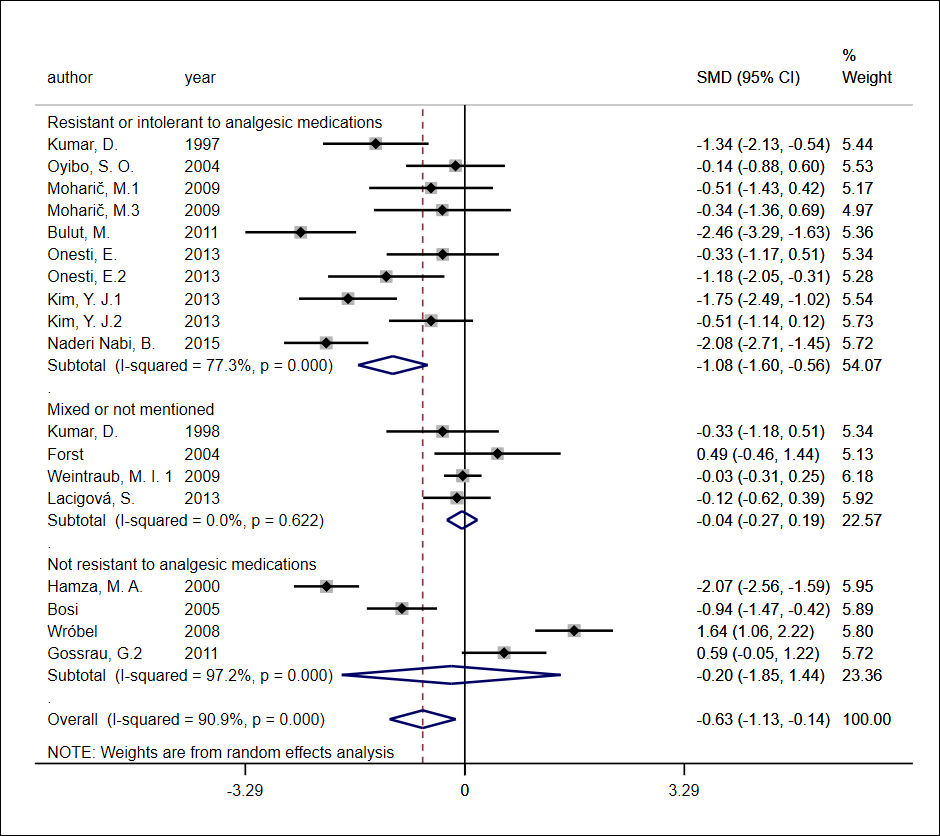


(D)Follow-up more than 1 month


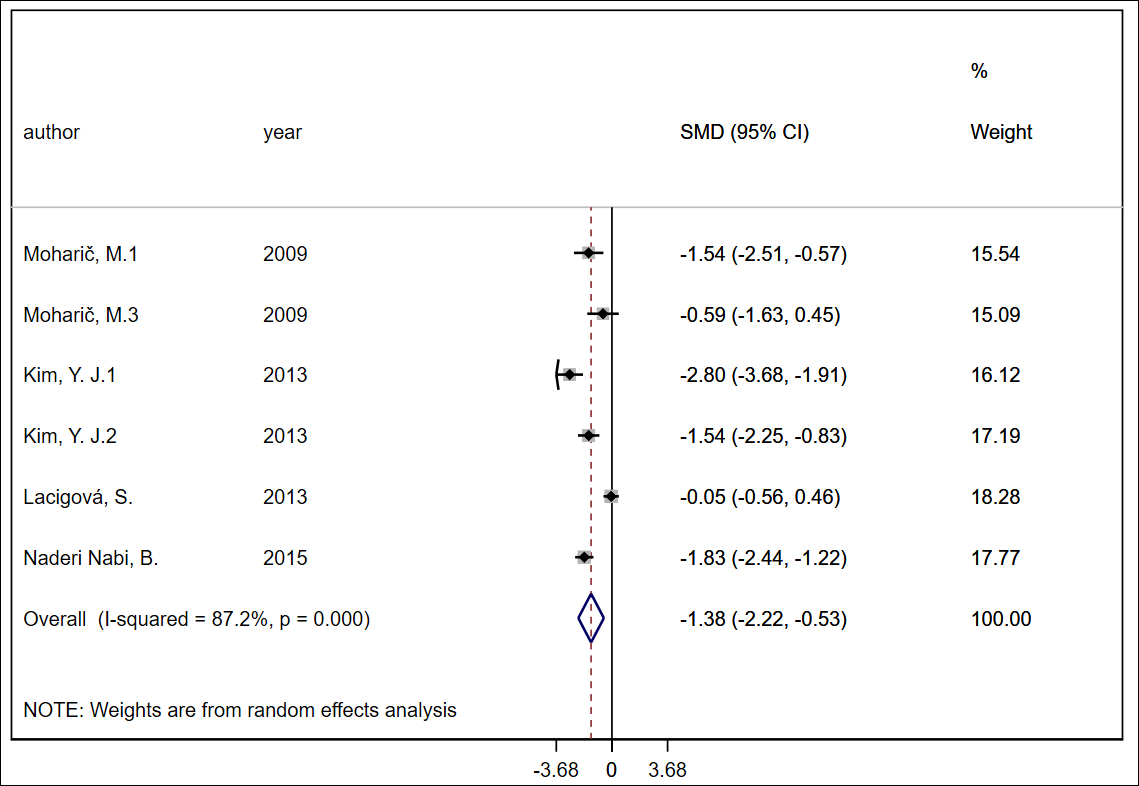


(E)Type of peripheral NINMs


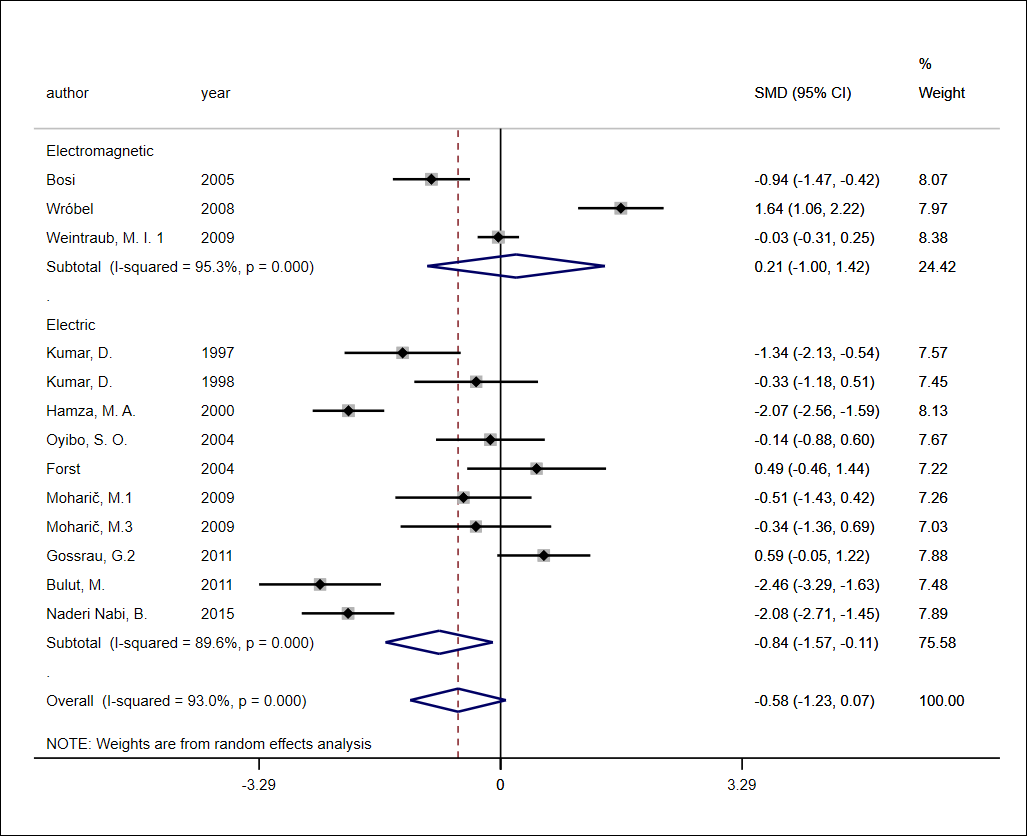


(F) Current Frequency of Peripheral Electric NINMs
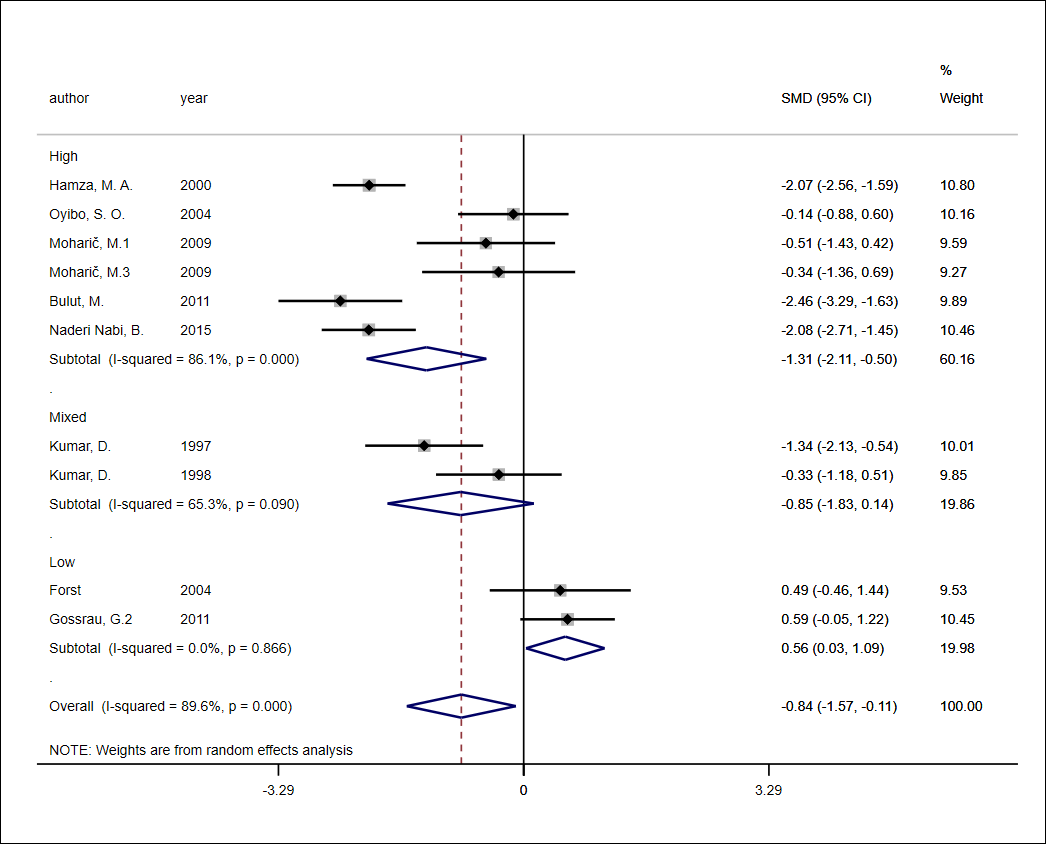


**Supplementary material 6: Funnel plot for pain score**


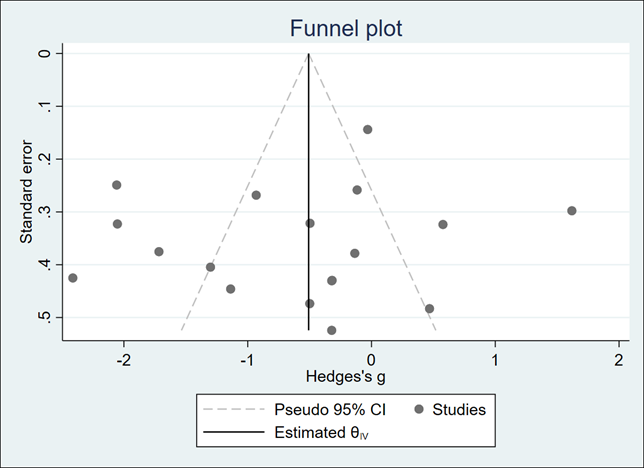

Supplement: Supplementary file 1 — Supplementary Information [file 41598_2020_75922_MOESM1_ESM.docx]
